# Left‐sided acute appendicitis with congenital gastrointestinal malrotation

**DOI:** 10.1002/jgh3.12804

**Published:** 2022-08-09

**Authors:** Van Trung Hoang, Hoang Anh Thi Van, The Huan Hoang, Tien Hoai Vo, Vichit Chansomphou

**Affiliations:** ^1^ Department of Radiology Thien Hanh Hospital Buon Ma Thuot Vietnam; ^2^ Department of Radiology Tam Tri Nha Trang General Hospital Nha Trang Vietnam; ^3^ Department of Radiology Savannakhet Medical‐Diagnostic Center Kaysone Phomvihane Laos

**Keywords:** clinical intestinal disorders, computed tomography, gastroenterology, imaging, intestinal disorders

## Abstract

We describe a 28‐year‐old man with acute appendicitis associated with gastrointestinal malrotation. The diagnosis was confirmed by a computed tomography scan, and he was treated by laparoscopic appendectomy without a Ladd procedure.

## Graphical abstract

Appendicitis in patients with gastrointestinal malrotation is a relatively rare condition. Imaging modalities, especially computed tomography scan, can assist in the accurate diagnosis of this entity. Treatment consists of appendectomy without or with a Ladd procedure.

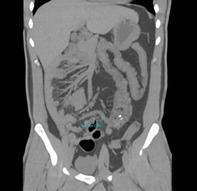

## Introduction

Gastrointestinal malrotations, while often associated with other birth anomalies, are an isolated feature in the majority of adult cases. The estimated incidence in live births of reported intestinal malrotation ranges from 0.03 to 0.5%. The true incidence in adults has not yet been precisely determined. Appendicitis in intestinal malrotation patients is a relatively rare surgical disease.^1^, ^2^

## Case report

**Figure 1:**
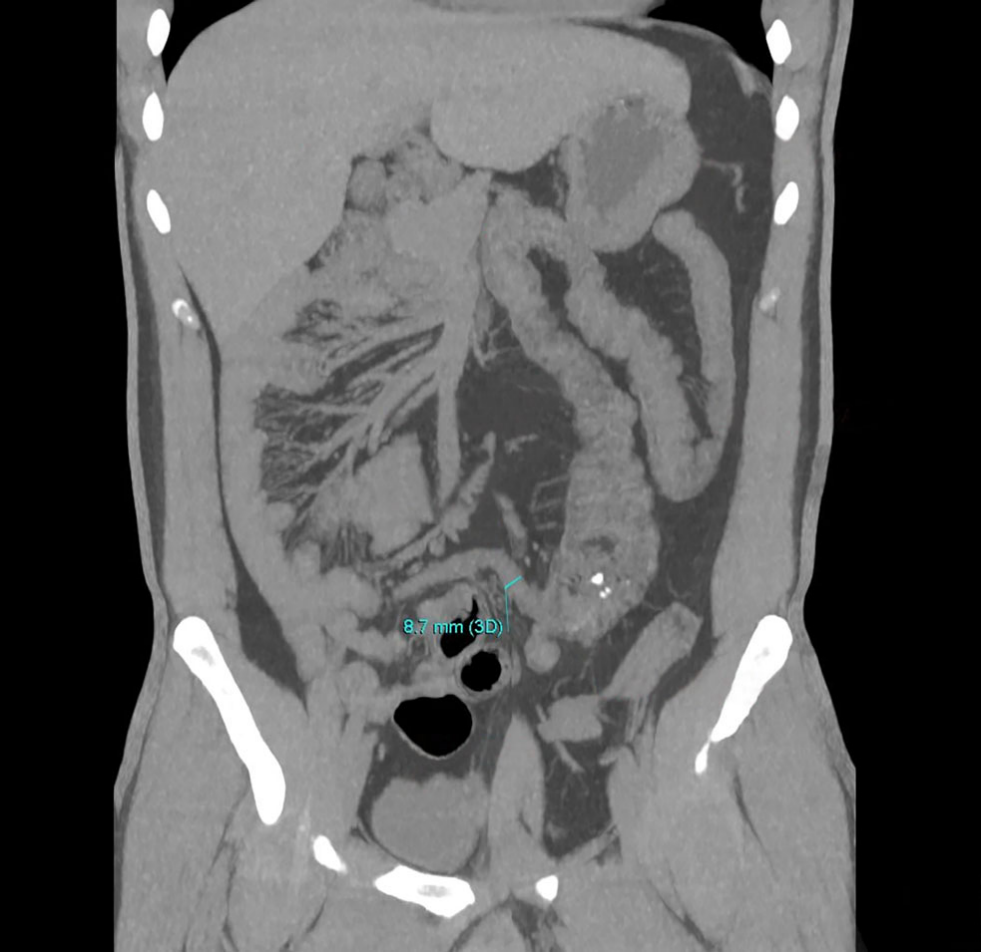
Coronal maximum intensity projection reconstruction computed tomography image showed dilated appendix in the left mid‐abdomen with congenital gastrointestinal malrotation. The appendix was located at the umbilicus with a diameter of about 8.5–10 mm, containing fluid inside and surrounding fat infiltration.

A 28‐year‐old man presented to the emergency department with a persistent dull abdominal pain for about 5 h. The patient stated that pain started in the epigastrium and then spread to the right iliac fossa. Additional symptoms included anorexia and nausea. The clinician suspected acute appendicitis and arranged for routine blood tests and an abdominal ultrasound study. He had an elevated white cell count (18 000/mL), but the ultrasound study failed to identify the appendix. Computed tomography (CT) scan performed subsequently confirmed the diagnosis of acute appendicitis with congenital gastrointestinal malrotation (Fig. 1). Subsequently, pain was largely located around the umbilicus and in the left iliac fossa. The patient was treated with laparoscopic appendectomy after a comprehensive preoperative evaluation without surgical intervention for intestinal malrotation. He was discharged from the hospital after 1 week and remained well after follow‐up for 2 years.

## Discussion

Gastrointestinal malrotation is a congenital anomaly defined as defective rotation of the middle intestine around the axis of the superior mesenteric artery. However, a variety of anatomical variants have been described.^1^ Appendicitis in an adult patient with gastrointestinal malrotation often presents with left‐sided abdominal pain that is misdiagnosed as diverticulitis in the absence of definitive imaging. Imaging modalities, especially CT, can easily and accurately diagnose this entity. CT plays a crucial role in pre‐surgical planning, accelerating the administration of definitive therapy, and redirecting the principal clinical team in some cases.^2^ At present, there are no clear guidelines on the surgical treatment of malrotation at the time of appendectomy. Surgical options for this situation include the Ladd procedure, which is a surgical intervention for intestinal malrotation encompassing volvulus detorsion, replacing the bowel in the abdomen with the small bowel in the right abdomen and the cecum in the left upper abdomen, Ladd's bands ligation, broadening the mesenteric base, and appendectomy. Open surgical intervention mostly takes precedence over laparoscopic surgery: it increases surgeons' comfort and reduces the risk of incomplete procedures, especially when approaching posterior duodenal attachments.^2^, ^3^

### Patient consent

Informed consent was obtained from the patient.
